# Association between autoimmune diseases and COVID-19 as assessed in both a test-negative case–control and population case–control design

**DOI:** 10.1186/s13317-020-00141-1

**Published:** 2020-10-06

**Authors:** Rossella Murtas, Anita Andreano, Federico Gervasi, Davide Guido, David Consolazio, Sara Tunesi, Laura Andreoni, Maria Teresa Greco, Maria Elena Gattoni, Monica Sandrini, Antonio Riussi, Antonio Giampiero Russo

**Affiliations:** Epidemiology Unit, Agency for Health Protection of Milan, Corso Italia 19, 20122 Milan, Italy

**Keywords:** Autoimmune diseases, Covid-19, Test-negative design

## Abstract

**Background:**

COVID-19 epidemic has paralleled with the so called infodemic, where countless pieces of information have been disseminated on putative risk factors for COVID-19. Among those, emerged the notion that people suffering from autoimmune diseases (AIDs) have a higher risk of SARS-CoV-2 infection.

**Methods:**

The cohort included all COVID-19 cases residents in the Agency for Health Protection (AHP) of Milan that, from the beginning of the outbreak, developed a web-based platform that traced positive and negative cases as well as related contacts. AIDs subjects were defined ad having one the following autoimmune disease: rheumatoid arthritis, systemic lupus erythematosus, systemic sclerosis, Sjogren disease, ankylosing spondylitis, myasthenia gravis, Hashimoto’s disease, acquired autoimmune hemolytic anemia, and psoriatic arthritis. To investigate whether AID subjects are at increased risk of SARS-CoV-2 infection, and whether they have worse prognosis than AIDs-free subjects once infected, we performed a combined analysis of a test-negative design case–control study, a case–control with test-positive as cases, and one with test-negative as cases (CC-NEG).

**Results:**

During the outbreak, the Milan AHP endured, up to April 27th 2020, 20,364 test-positive and 34,697 test-negative subjects. We found no association between AIDs and being positive to COVID-19, but a statistically significant association between AIDs and being negative to COVID-19 in the CC-NEG. If, as likely, test-negative subjects underwent testing because of respiratory infection symptoms, these results imply that autoimmune diseases may be a risk factor for respiratory infections in general (including COVID-19), but they are not a specific risk factor for COVID-19. Furthermore, when infected by SARS-CoV-2, AIDs subjects did not have a worse prognosis compared to non-AIDs subjects. Results highlighted a potential unbalance in the testing campaign, which may be correlated to the characteristics of the tested person, leading specific frail population to be particularly tested.

**Conclusions:**

Lack of availability of sound scientific knowledge inevitably lead unreliable news to spread over the population, preventing people to disentangle them form reliable information. Even if additional studies are needed to replicate and strengthen our results, these findings represent initial evidence to derive recommendations based on actual data for subjects with autoimmune diseases.

## Background

Since its first appearance in China in December 2019, and even more with its worldwide subsequent diffusion, COVID-19 epidemic has paralleled with the so called infodemic. Countless pieces of information (often lacking scientific validity) have been disseminated on putative risk factors for COVID-19 trough traditional and social media channels [1]. Among this information, the notion that people suffering from autoimmune diseases (AIDs) have a higher risk of SARS-CoV-2 infection emerged. In fact, one can speculate that subjects with AIDs might be at greater risk of infections for the AID itself, but also because of immunomodulatory treatment and secondary chronic conditions. In literature, subjects with rheumatoid arthritis (RA) have been found to have higher risk of death from infections [2–4] and higher risk of nonfatal infections [5, 6] compared to the general population. Tektonidou et al. [7] found an increased risk of hospitalization for serious infections in subjects with systemic lupus erythematosus (SLE) and Bosch et al. [8] concluded that subjects with SLE have an increased overall risk for infections (including pneumonia). Also, an increased risk of pneumonia from different coronavirus infection has been reported in immunocompromised subjects [9].

However, limited scientific evidence is currently available on the association between AIDs and COVID-19. Articles on the subject, published in peer-reviewed medical journals, are mostly general recommendations or systemic reviews providing an overview on viral infectious risk in AIDs subjects [10]. In a literature review, Favalli et al. [11], hypothesized a two-way association between rheumatoid arthritis (RA) and COVID-19: microorganisms can indeed produce acute and chronic arthritis through direct colonisation of the joints or inducing an autoimmune response to the infection. At the same time, an increase in risk of infection in RA subjects compared to the general population due to the impairment of the immune system typical of autoimmune disorders is well documented: Askanase et al. [12] pointed out a lack of knowledge in the COVID-related respiratory complications in subjects with autoimmune diseases, in particular for SLE subjects that may be susceptible to the more severe manifestations of COVID-19, such as pneumonia. They even suggested that high type I interferon levels, found clustered in SLE families [13], may exert, on the contrary, a protective effect on COVID-19. Few studies have been focusing on quantifying the relationship between AIDs and susceptibility to SARS-COV-2 infection or to severe COVID-19 disease. D’Silva et al. [14] investigated differences in manifestations and outcomes of coronavirus disease 2019 infection between subjects with rheumatic disease (RD) and subjects without RDs. They found similar characteristic between RD and non-RD subjects on hospitalizations, and significantly different prognosis in RD subjects requiring more often intensive care admission and mechanical ventilation. Liu et al. [15], through a meta-analysis, showed that AID was associated with a 1.21-fold increased risk of severe COVID-19 disease and with a 1.31-fold increased risk of mortality in subjects with COVID-19. However, no details on which diseases were considered was available, and none of the found increases in risk was statistically significant overall or by country. In a second study, Emmi et al. [16], in a sample of subjects with AID residing in Tuscany, found a prevalence of COVID-19 comparable to that observed in the general population of Tuscany.

Most of the available studies attributed the potential association between AID and susceptibility to COVID-19, infection or severe disease, to immunosuppressive or immunomodulatory therapies used to treat AIDs [17–19]. Subjects treated with high-dose corticosteroids are overall considered at significant risk of serious infection [20, 21]. On the other hand, conventional disease-modifying anti-rheumatic drugs are not considered a risk factor for COVID-19 [21, 22], and some immunosuppressive medications (such as tocilizumab), have been found effective in alleviating symptoms and even recommended for severe COVID-19 management in addition to standard therapy [23].

In this work, we aim to investigate whether AID subjects are at increased risk of SARS-CoV-2 infection, and whether they have worse prognosis than AIDs-free subjects once infected.

## Methods

### Data sources

The cohort included all COVID-19 cases in the study area, covered by the Agency for Health Protection (AHP) of Milan, corresponding to 193 municipalities in the northern Italian region of Lombardy, with a total population of 3.48 million inhabitants. From the beginning of the outbreak, all tracing activities were included in a web-based platform, developed by the Epidemiologic Unit of the AHP, called Milano COV, including cases and related close contacts. A confirmed-case is defined as a person with a real-time polymerase chain reaction (RT-PCR) positive result of SARS-COV-2 infection, irrespective of clinical signs and symptoms. Cases and close contacts underwent epidemiological investigation to provide description of the clinical presentation of COVID-19 and its clinical course.

Additional information relating to hospitalizations of patients was derived from the regular administrative data discharge flow, which is consolidated for hospital admissions up to the end of April 2020. We integrated between them, and verified with the demographic information in the Health Service Register of the Lombardy Region (age, gender, place of residence), the described data sources in the Integrated Data warehouse for COVID Analysis in Milan, anonymized with a random unique patient id. The same id was assigned to those subjects in all other administrative databases of the AHP, anonymized prior to analysis. Individual level comorbidities data were derived using the chronic disease administrative database of the AHP of Milan, according to the algorithms specified in the Regional Act X/6164 [24] and X/7655 [25] of 2017, and summarized in English in Additional file 1. Vital status was derived from the early notification system of the AHP of Milan, set-up from the beginning of the epidemic, in which deaths are communicated from the Civil Registry of each Municipality to the AHP and manually introduced in the Health Service Register, or directly from the general practitioner (GP) and Mayor’s. We determined vital status at 30-day from diagnosis, which was defined for confirmed-cases as the first date between registered symptom onset and the swab positivity result. The date of symptom onset in the database was derived from the epidemiological interview or from the date of first access to an emergency department or first thorax CT scan, in this order of priority. If none of these dates was available and the patient had been hospitalized, the date of hospital admission was used. For a minority of patients, infected in the early phase of the epidemic and for whom no onset dates were available, we uniformly random imputed the date of symptoms onset between February 10th and 17th. For this analysis, we considered as alive patients with a date of death more than 30 days after the date of diagnosis. The vital status was assessed on May 23th 2020.

**Table 1:** Overall demographic and clinical characteristics of test-positive and test-negative subjects, and population controls (for the case–control design 2) by autoimmune status

	Test-positive subjects			Test-negative subjects			Controls (for case–control design 2)		
	Overall	Autoimmune status		Overall	Autoimmune status		Overall	Autoimmune status	
		No	Yes		No	Yes		No	Yes
	n = 20,364	n = 19,699 96.7%	n = 665 3.3%	n = 34,697	n = 33,400 96.3%	n = 1297 3.7%	n = 81,414	n = 79,245 97.3%	n = 2169 2.7%
Gender = Male (%)	9666 (47.5)	9496 (48.2)	170 (25.6)	13,658 (39.4)	13,438 (40.2)	220 (17)	38,659 (47.5)	38,127 (48.1)	532 (24.5)
Age mean (sd)	65.5 (19.4)	65.6 (19.5)	64.5 (16.1)	54.8 (20.8)	54.8 (20.95)	56.9 (16.4)	65.5 (19.4)	65.5 (19.5)	66.2 (16.1)
Age class (years) (%)									
< 17	171 (0.8)	170 (0.9)	1 (0.2)	905 (2.6)	902 (2.7)	3 (0.2)	684 (0.8)	682 (0.9)	2 (0.1)
[18–40)	1907 (9.4)	1868 (9.5)	39 (5.9)	7521 (21.7)	7353 (22)	168 (13)	7629 (9.4)	7512 (9.5)	117 (5.4)
[40–70)	8622 (42.3)	8264 (42)	358 (53.8)	17,047 (49.1)	16,241 (48.6)	806 (62.1)	34,488 (42.4)	33,452 (42.2)	1036 (47.8)
≥ 70	9664 (47.5)	9397 (47.7)	267 (40.2)	9224 (26.6)	8904 (26.7)	320 (24.7)	38,613 (47.4)	37,599 (47.4)	1014 (46.7)
Geographic location (%)									
Lodi province (start of the outburst)	2939 (14.4)	2836 (14.4)	103 (15.5)	5185 (14.9)	5001 (15)	184 (14.2)	11,675 (14.3)	11,356 (14.3)	319 (14.7)
City of Milan	7235 (35.5)	7025 (35.7)	210 (31.6)	13,188 (38)	12,761 (38.2)	427 (32.9)	29,185 (35.8)	28,437 (35.9)	748 (34.5)
Milan province	10,190 (50)	9838 (49.9)	352 (52.9)	16,324 (47)	15,638 (46.8)	686 (52.9)	40,554 (49.8)	39,452 (49.8)	1102 (50.8)
Setting (%)									
Home	6122 (30.1)	5861 (29.8)	261 (39.2)	–	–	–	–	–	–
Residential	3381 (16.6)	3319 (16.8)	62 (9.3)	–	–	–	–	–	–
Residential followed by hospitalization	556 (2.7)	548 (2.8)	8 (1.2)	–	–	–	–	–	–
Hospitalized	10,305 (50.6)	9971 (50.6)	334 (50.2)	–	–	–	–	–	–
Health status									
Non-hospitalized and alive	8292 (40.7)	7998 (40.6)	294 (44.2)	–	–	–	–	–	–
Hospitalized and alive	7949 (39.0)	7692 (39.0)	257 (38.6)	–	–	–	–	–	–
Deceased	4123 (20.2)	4009 (20.4)	114 (17.1)	–	–	–	–	–	–
Number of comorbidities (Excluded autoimmune diseases)									
None	8265 (40.6)	8105 (41.1)	160 (24.1)	18,870 (54.4)	18,457 (55.3)	413 (31.8)	36,658 (45)	36,131 (45.6)	527 (24.3)
1–3	8951 (44)	8581 (43.6)	370 (55.6)	11,959 (34.5)	11,286 (33.8)	673 (51.9)	36,156 (44.4)	34,862 (44)	1294 (59.7)
≥ 4	3148 (15.5)	3013 (15.3)	135 (20.3)	3868 (11.1)	3657 (10.9)	211 (16.3)	8600 (10.6)	8252 (10.4)	348 (16)
Comorbidities (%)									
Transplanted any time = Yes (%)	77 (0.4)	75 (0.4)	2 (0.3)	227 (0.7)	221 (0.7)	6 (0.5)	159 (0.2)	155 (0.2)	4 (0.2)
Blood and Hematopoietic organs = Yes (%)	30 (0.1)	27 (0.1)	3 (0.5)	64 (0.2)	57 (0.2)	7 (0.5)	85 (0.1)	80 (0.1)	5 (0.2)
HIV infection or AIDS = Yes (%)	72 (0.4)	72 (0.4)	0 (0)	224 (0.6)	220 (0.7)	4 (0.3)	261 (0.3)	259 (0.3)	2 (0.1)
Tumor in first line treatment = Yes (%)	1024 (5)	923 (4.7)	101 (15.2)	2027 (5.8)	1869 (5.6)	158 (12.2)	3303 (4.1)	3023 (3.8)	280 (12.9)
Tumor in follow-up, 1–5 years = Yes (%)	788 (3.9)	759 (3.9)	29 (4.4)	1128 (3.3)	1071 (3.2)	57 (4.4)	3153 (3.9)	3050 (3.8)	103 (4.7)
Tumor in remission after 5 years = Yes (%)	1087 (5.3)	1038 (5.3)	49 (7.4)	1337 (3.9)	1269 (3.8)	68 (5.2)	4238 (5.2)	4088 (5.2)	150 (6.9)
Type 1 diabetes = Yes (%)	28 (0.1)	28 (0.1)	0 (0)	79 (0.2)	76 (0.2)	3 (0.2)	58 (0.1)	54 (0.1)	4 (0.2)
Type 2 diabetes = Yes (%)	2426 (11.9)	2357 (12)	69 (10.4)	2537 (7.3)	2434 (7.3)	103 (7.9)	7660 (9.4)	7433 (9.4)	227 (10.5)
Complicated DM Type 1 and 2 = Yes (%)	423 (2.1)	408 (2.1)	15 (2.3)	527 (1.5)	503 (1.5)	24 (1.9)	750 (0.9)	722 (0.9)	28 (1.3)
Hypercholesterolaemia = Yes (%)	2301 (11.3)	2216 (11.2)	85 (12.8)	2614 (7.5)	2495 (7.5)	119 (9.2)	9607 (11.8)	9306 (11.7)	301 (13.9)
Arterial hypertension = Yes (%)	8187 (40.2)	7896 (40.1)	291 (43.8)	9454 (27.2)	9016 (27)	438 (33.8)	32,202 (39.6)	31,267 (39.5)	935 (43.1)
Ischemic heart disease = Yes (%)	2364 (11.6)	2297 (11.7)	67 (10.1)	2744 (7.9)	2617 (7.8)	127 (9.8)	7497 (9.2)	7273 (9.2)	224 (10.3)
Valvular heart disease = Yes (%)	475 (2.3)	458 (2.3)	17 (2.6)	678 (2)	647 (1.9)	31 (2.4)	1328 (1.6)	1287 (1.6)	41 (1.9)
Cardiomyopathy with arrhythmia = Yes (%)	2310 (11.3)	2241 (11.4)	69 (10.4)	2614 (7.5)	2503 (7.5)	111 (8.6)	6850 (8.4)	6634 (8.4)	216 (10)
Cardiomyopathy without arrhythmia = Yes (%)	1699 (8.3)	1641 (8.3)	58 (8.7)	2025 (5.8)	1941 (5.8)	84 (6.5)	5278 (6.5)	5129 (6.5)	149 (6.9)
Chronic hearth failure = Yes (%)	1401 (6.9)	1346 (6.8)	55 (8.3)	1764 (5.1)	1680 (5)	84 (6.5)	3567 (4.4)	3463 (4.4)	104 (4.8)
Peripheral artery disease = Yes (%)	595 (2.9)	573 (2.9)	22 (3.3)	820 (2.4)	784 (2.3)	36 (2.8)	1334 (1.6)	1290 (1.6)	44 (2)
Venous diseases = Yes (%)	201 (1)	192 (1)	9 (1.4)	281 (0.8)	259 (0.8)	22 (1.7)	580 (0.7)	562 (0.7)	18 (0.8)
Cerebrovascular disease = Yes (%)	822 (4)	799 (4.1)	23 (3.5)	932 (2.7)	884 (2.6)	48 (3.7)	1653 (2)	1607 (2)	46 (2.1)
Thyroid diseases = Yes (%)	1137 (5.6)	783 (4)	354 (53.2)	2149 (6.2)	1357 (4.1)	792 (61.1)	4106 (5)	2872 (3.6)	1234 (56.9)
Other endocrine diseases = Yes (%)	78 (0.4)	70 (0.4)	8 (1.2)	173 (0.5)	150 (0.4)	23 (1.8)	225 (0.3)	211 (0.3)	14 (0.6)
Other autoimmune diseases = Yes (%)	359 (1.8)	22 (0.1)	337 (50.7)	601 (1.7)	47 (0.1)	554 (42.7)	1095 (1.3)	63 (0.1)	1032 (47.6)
Epilepsy = Yes (%)	271 (1.3)	257 (1.3)	14 (2.1)	393 (1.1)	380 (1.1)	13 (1)	598 (0.7)	577 (0.7)	21 (1)
Alzheimer and dementias = Yes (%)	669 (3.3)	655 (3.3)	14 (2.1)	579 (1.7)	562 (1.7)	17 (1.3)	989 (1.2)	968 (1.2)	21 (1)
Parkinson and parkinsonisms = Yes (%)	310 (1.5)	305 (1.5)	5 (0.8)	287 (0.8)	279 (0.8)	8 (0.6)	616 (0.8)	596 (0.8)	20 (0.9)
Nervous system diseases, others = Yes (%)	99 (0.5)	94 (0.5)	5 (0.8)	232 (0.7)	218 (0.7)	14 (1.1)	263 (0.3)	257 (0.3)	6 (0.3)
Chronic hepatitis and cirrhosis = Yes (%)	433 (2.1)	413 (2.1)	20 (3)	705 (2)	666 (2)	39 (3)	1293 (1.6)	1248 (1.6)	45 (2.1)
Digestive system diseases, others = Yes (%)	254 (1.2)	243 (1.2)	11 (1.7)	459 (1.3)	434 (1.3)	25 (1.9)	763 (0.9)	710 (0.9)	53 (2.4)
COPD = Yes (%)	897 (4.4)	864 (4.4)	33 (5)	1089 (3.1)	1039 (3.1)	50 (3.9)	2433 (3)	2345 (3)	88 (4.1)
RF or oxygen therapy = Yes (%)	97 (0.5)	91 (0.5)	6 (0.9)	151 (0.4)	142 (0.4)	9 (0.7)	221 (0.3)	207 (0.3)	14 (0.6)
Asthma = Yes (%)	443 (2.2)	422 (2.1)	21 (3.2)	833 (2.4)	776 (2.3)	57 (4.4)	1369 (1.7)	1303 (1.6)	66 (3)
CKD = Yes (%)	590 (2.9)	557 (2.8)	33 (5)	716 (2.1)	681 (2)	35 (2.7)	1464 (1.8)	1409 (1.8)	55 (2.5)
Dialysis dependent CKD = Yes (%)	151 (0.7)	147 (0.7)	4 (0.6)	443 (1.3)	427 (1.3)	16 (1.2)	122 (0.1)	121 (0.2)	1 (0)
Autoimmune diseases									
Acquired autoimmune hemolytic anemia	–	–	10 (1.5)	–	–	11 (0.8)	–	–	19 (0.9)
Systemic sclerosis	–	–	21 (3.2)	–	–	47 (3.6)	–	–	51 (2.4)
Ankylosing spondylitis	–	–	13 (2)	–	–	22 (1.7)	–	–	42 (1.9)
Myasthenia gravis	–	–	20 (3)	–	–	21 (1.6)	–	–	39 (1.8)
Rheumatoid arthritis	–	–	167 (25.1)	–	–	223 (17.2)	–	–	479 (22.1)
Psoriatic arthritis	–	–	69 (10.4)	–	–	139 (10.7)	–	–	284 (13.1)
Systemic lupus erythematosus	–	–	16 (2.4)	–	–	38 (2.9)	–	–	57 (2.6)
Sjögren disease	–	–	26 (3.9)	–	–	60 (4.6)	–	–	70 (3.2)
Hashimoto’s disease	–	–	323 (48.6)	–	–	736 (56.7)	–	–	1128 (52.0)

### Study population

From the COVID-19 database of the Milan AHP we extracted, on April 27, 2020, all subjects with positive and negative SARS-COV-2 nasopharyngeal swabs. Henceforth, subjects with positive SARS-COV-2 nasopharyngeal swabs will be called test-positives and subjects with negative SARS-COV-2 nasopharyngeal swabs will be called test-negative subjects. Through the database, we collected demographic information on age, gender, municipality of residence, ASST (geographical and administrative partition of the territory of the Milan AHP). We defined as exposed all subjects with the following autoimmune diseases: AR, SLE, systemic sclerosis, Sjogren disease, ankylosing spondylitis, myasthenia gravis, Hashimoto’s disease, acquired autoimmune hemolytic anemia, and psoriatic arthritis. Presence of any autoimmune disease was identified in the chronic disease administrative database of the AHP of Milan, where information on the presence of 64 chronic conditions is recorded, for every resident registered with the RHS, using outpatient exams and visits, hospital discharge sheets, pharmaceutical, and exemption from co-payment databases according to the algorithms defined in the Regional Act X/6164 and X/7655 of 2017 [24, 25]. Number of comorbidities was derived from the same database. For test-positive subjects, death and hospitalization status were updated to June 11, 2020.

### Study design

To evaluate the association between autoimmune status and occurrence of COVID-19 we performed a combined analysis of a test-negative design (TND) case–control study, a case–control with test-positive as cases (CC-POS design), and one with test-negative as cases (CC-NEG design). As proposed by Vandenbroucke et al. [26], the combination of these studies will serve to evaluate if autoimmune diseases are specific risk factors for COVID-19 or generally for respiratory diseases with similar symptoms. We also performed a conventional matched case–control design for comparison.

TNDs evaluate the association between an exposure and an outcome by comparing test-positive (cases) with test-negative (controls) subjects. TND cases were defined as all subjects with a positive swab collected from the *ATS*-*Milano COV* system, no exclusions were performed. TND controls were all subjects with a negative swab included in the same database. The idea is that test-negative controls underwent testing because they presented symptoms attributable to COVID-19 but resulted negative, thus having a different infection which may lead to similar symptoms, for example another respiratory infection. In fact, being susceptible to the same selection mechanisms as tested-positives will protect from common case–control biases. The TND design is potentially capable of identifying the effect of autoimmunity on COVID-19 if it has a different magnitude, or even direction, compared to the effect that it has in other respiratory infections [26]. To control for different spatial correlation among subjects, and to control for time trends in the administration of swabs, we matched TND’s cases and controls by ASST and date of swab, date of positive swab for cases and date of negative swab for controls within 7 days of the case index date, only matched cases and controls will be considered.

At the same time, comparing test-positive subjects with general population controls will allow to estimate the effect of autoimmunity on COVID-19 infection compared to a control without respiratory symptoms. On the other hand, comparing test-negative subjects to the general population will allow to assess if autoimmunity is a risk factor for respiratory infections in general. Consequently, we designed two additional case–controls: the CC-POS and CC-NEG designs. In the CC-POS the cases were those subjects with a positive swab while in the CC-NEG the cases were those subjects with a negative swab collected from the *ATS*-*Milano COV* system. Test-negative subjects (the cases in the CC-NEG design) were the same subjects selected as controls in the TND design [26]. In order to compare CC-POS’s (and CC-NEG’s) results with TND, a number of controls equal to 4 times the number of test-positive plus test-negative subjects was randomly sampled from the general population of the Milan AHP. Thus, controls for both the CC-POS and CC-NEG design were identical.

For comparison, we performed also a classical population case–control design (hereafter name case–control design 2), where cases (test-positive subjects) and controls are matched by age (± 5 years), gender and municipality of residence. Controls were randomly sampled from the general population of the Milan AHP, also with a ratio of 1:4, only cases matched with 4 controls will be considered.

To evaluate the association between autoimmune status and a proxy of disease severity, defined as non-hospitalized and alive, hospitalized and alive, and deceased, we performed a cohort study using all COVID-19 test-positive subjects.

### Statistical analysis

To measure the association between autoimmune diseases and COVID-19 occurrence we used logistic regression models and conditional logistic regression models in matched designs, presenting results as the ORs of having an autoimmune disease in cases compared to controls and their 95% CIs.

Models for the TND design were adjusted for age (categorized as < 17, [18–40), [40–70), ≥ 70 years), gender and number of non-AIDs chronic conditions (categorized as no conditions, 1–3, and ≥ 4). Models for CC-POS and CC-NEG were adjusted for gender, age (categorized as < 17, [18–40), [40–70), ≥ 70 years), number of non-AIDs chronic conditions (categorized as no conditions, 1-3, and ≥ 4) and municipality of residence. Models for the classical Population Case–Controls Design were adjusted for number of non-AIDs chronic conditions (categorized as no conditions, 1–3, and ≥ 4).

To measure the association between autoimmune diseases and severity of COVID-19 disease we used ordinal (cumulative) and multinomial logistic models. Ordinal logistic regression, in its cumulative formulation, requires the assumption of proportional odds [27]. When any covariate did not satisfy this assumption, a partial proportional odds model was fitted allowing non-proportionality for the selected variables [28]. Ordinal and multinomial logistic models were adjusted for gender, age (categorized as < 17, [18–40), [40–70), ≥ 70 years), number of non-AIDs chronic conditions (categorized as no conditions, 1–3, and ≥ 4) and ASST (n = 6). We decided to adjust for ASST, and not for municipality, of residence given that there are 193 municipality in the territory of the AHP, which may have led to very few cases in each stratum. Results were displayed as ORs with 95% CIs. ORs for the ordinal logistic model will be interpreted in their cumulative formulation that is the odds of deceased versus the combined categories hospitalized and alive, and non-hospitalized and alive, and of the combined categories deceased, and hospitalized and alive versus non-hospitalized and alive. ORs for the multinomial logistic regression will be interpreted as usual ORs with non-hospitalized and alive as the reference category. The analyses were performed using SAS Software version 9.4 (SAS Institute Inc., Cary, NC, USA).

## Results

### Description of cases and controls

During the outbreak, the Milan AHP endured, up to April 27th 2020, 20364 test-positive and 34697 test-negative subjects (overall demographic and clinical characteristics are reported in Table 1). Demographic and clinical characteristics of test-negative matched to test-positive subjects by ASST and date of swab, and of population controls for CC-POS and CC-NEG designs are reported in Additional file 2.

The proportion of males among tested-positives was 47.5%, similar to population controls (47.5% for case–control design 2 and 48.4% for the CC-POS and CC-NEG designs) and higher than test-negative subjects (39.4% overall, and 40.7% after matching). The majority of tested-positives had more than 70 years (47.5%), as well as population controls for the case–control design 2 (47.4%). On the contrary, the proportion of people older than 70 years was lower in test-negative subjects (26.6% overall, 26.1% after matching) and controls for the CC-POS and CC-NEG designs (18.1%). However, mean age in test-negative subjects was 54.8 years (s.d. 20.8, Table 1) in the overall test-negative group and 54.8 (s.d. 20.6) in the matched test-negative group (Additional file 2), higher than in the random sample of the general population controls used for CC-POS and CC-NEG where the mean age was 45.4 years (s.d. 23.7, Additional file 2).

Most of cases presented at least one non-AIDs comorbidity (60%), and 15.5% of tested-positives had at least 4 non-AIDs comorbidities. Among test-negative subjects, the number of non-AIDs comorbidities was quite different, with the majority of people having no comorbidities (54.4%) and approximately 45% having at least one non-AIDs comorbidity. Only 32% of the general population controls used for CC-POS and CC-NEG had at least one non-AIDs comorbidity while the majority of controls for the case–control design 2 had at least one comorbidity (55%).

Among tested-positives, 665 (3.2%) had an AID disease with the majority having Hashimoto’s thyroiditis (48.6%) followed by rheumatoid arthritis (25.1%). The figures were slightly higher in test-negative subjects, with 1297 (3.9%) having an autoimmune disease, 56.7% having Hashimoto’s thyroiditis and 17.2% having rheumatoid arthritis. The proportions of AIDs subjects among population controls (case–control design 2) were considerably different compared to test-positive and test-negative subjects, with 2169 persons with an autoimmune disease (2.7%), most of them again having Hashimoto’s thyroiditis (52%) followed by rheumatoid arthritis (22.1%).

Almost 41% of test-positive subjects were non-hospitalized and alive, 39% were hospitalized and alive, and 20% were deceased.

### Autoimmune disease and COVID-19 occurrence

The adjusted OR of having an autoimmune disease in COVID-19 test-positive compared to test-negative subjects was 0.86 (95% CI 0.76–0.96) in the TND design analysis (Table 2). Comparing test-positive subjects to a random sample of population controls (CC-POS), the unadjusted OR of having an autoimmune disease was 1.45 (95% CI 1.32–1.58) which shrunken to no-association when adjusted by covariates (OR = 0.98, 95% CI 0.90–1.08). On the other hand, the adjusted OR of having an autoimmune disease was 1.19 (1.09–1.29) for test-negative subjects compared to a random sample of population controls (CC-NEG).

**Table 2:** Un-adjusted and adjusted odds ratios for COVID-19 according to autoimmune disease from case–control designs

Designs	Exposure		OR (95% CI)	
			Un-adjusted	Adjusted
Test-negative case–control^a^	Autoimmune status	No	Referent	Referent
		Yes	0.86 (0.77–0.96)	0.90 (0.80–1.01)
Positive case–control (CC-POS)^a^	Autoimmune status	No	Referent	Referent
		Yes	1.51 (1.38–1.64)	1.00 (0.92–1.10)
Negative case–control (CC-NEG)^a^	Autoimmune status	No	Referent	Referent
		Yes	1.75 (1.62–1.90)	1.15 (1.05–1.25)
Population case–control (case–control design 2)^b^	Autoimmune status	No	Referent	Referent
		Yes	1.24 (1.13–1.35)	1.16 (1.06–1.26)

^a^Odds ratios and corresponding 95% confidence intervals were calculated from a multivariable logistic model adjusted for gender, age (categorized as < 17, [18–40), [40–70), ≥ 70 years), number of non-autoimmune chronic conditions (categorized as no conditions, 1–3, ≥ 4), and municipality of residence (for CC-POS and CC-NEG). Test-positive subjects were matched to test-negatives by ASST and date, date of positive swab for cases and date of negative swab for controls within 7 days of the case index date

^b^Odds ratios and corresponding 95% confidence intervals were calculated from a conditional multivariable logistic model adjusted for number of non-autoimmune chronic conditions (categorized as no conditions, 1–3, ≥ 4); cases and controls matched by gender, age and municipality of residence

In the case–control design 2, matching for age, gender and municipality of residence (among 20,364 test-positive subjects, 20,327 where matched with 4 controls), we found a positive association between being diagnosed with COVID-19 and autoimmune disease, with an adjusted OR of having an autoimmune disease in tested-positives compared to controls of 1.16 (95% CI 1.06–1.26).

### Autoimmune disease and COVID-19 severity

Among autoimmune subjects, 44% were non-hospitalized and alive, and 17.1% died (Table 1). Treating the proxy variable of disease severity as ordinal, we found no association between having a more severe outcome and having an autoimmune disease, with an adjusted OR of 0.96 (95% CI 0.83–1.12), which is the odds of deceased versus the combined categories hospitalized and alive, and non-hospitalized and alive, and of the combined categories deceased, and hospitalized and alive versus non-hospitalized and alive (Table 3). Similar results were obtained using multinomial logistic regression, the adjusted OR of having an autoimmune disease was 1.05 (95% CI 0.88–1.25) for the group being hospitalized and alive compared to non-hospitalized and alive, and 0.95 (95% CI 0.74–1.22) for the group being deceased compared to non-hospitalized and alive.

**Table 3:** Un-adjusted and adjusted odds ratios for health status among test-positive subjects according to autoimmune disease from ordinal and multinomial logistic models

Model	Health status	Exposure		Un-adjusted OR (95% CI)	Adjusted OR (95% CI)
Ordinal Logistic Model^a,b^	–	Autoimmune status	No	Referent	Referent
			Yes	0.85 (0.73–0.98)	0.96 (0.83–1.12)
Multinomial Logistic Model^a^	Being hospitalized and alive vs non hospitalized and alive	Autoimmune status	No	Referent	Referent
			Yes	0.91 (0.77–1.08)	1.05 (0.88–1.25)
	Being deceased vs non hospitalized and alive (95% CI)	Autoimmune status	No	Referent	Referent
			Yes	0.77 (0.62–0.96)	0.95 (0.74–1.22)

Health status was defined as non-hospitalized and alive (n = 8451, 41.5%), hospitalized and alive (n = 8073, 39.6%), and deceased (n = 3840, 18.9%)

^a^Odds ratios and corresponding 95% confidence intervals were calculated from logistic models adjusted for gender, age (categorized as < 17, [18–40), [40–70), ≥ 70 years), number of non-autoimmune chronic conditions (categorized as no conditions, [1–4), ≥ 4), and ASST

^b^Age, gender and ASST did not satisfy the proportional odds assumption thus they have been inserted as having unequal slopes in the ordinal logistic regression model

## Discussion

In this work, we evaluated the association between autoimmune disease and risk of COVID-19 in the population of the AHP of Milan, an area particularly damaged by the virus, which includes the municipality of Codogno (where the Italian outbreak started). Concerning autoimmunity and COVID-19, we found a negative association between autoimmune status and risk of COVID-19 for tested-positives compared to tested-negatives controls. When comparing test-positive and test-negative subjects with a random sample of the population (by CC-POS and CC-NEG designs), we found no association between the exposure and a positive swab result, but a statistically significant association between the exposure and a negative swab result. If we are willing to assume that test-negative subjects underwent testing because of different disease but presented symptoms attributable to COVID-19, such as another respiratory infection, these results seem to imply that autoimmune diseases may be a risk factor for respiratory infections in general (including COVID-19), but they might not be a specific risk factor for COVID-19. On the other hand, the higher proportion of autoimmune diseases in the tested population compared to non-tested population (3.2% in test-positives, 3.9% in test-negatives, and 2.7% or 2.2% in the general population) highlights a potential unbalance of the exposure in the testing campaign, that is AIDs subjects were more likely to get tested than general population. However, comparing test-positives with the general population (by case–control design 2), we found that autoimmune disease is a risk factor for COVID-19. Vandenbroucke et al. [26] suggested that, when testing campaigns are correlated to the characteristics of the tested person, or general practitioners may serve specific frail populations, usual case–controls designs could produce biased results given that tested subjects might be self-selected contrary to the underlying population. In fact, the results had shown that test-negative subjects were older and had higher proportions of comorbidities compared to general population. In addition, the results suggested that AIDs subjects, when infected by SARS-CoV-2, do not have a worse prognosis compared to non-AIDs subjects.

Being one of the first study aimed at investigating the association between autoimmune diseases and COVID-19, the present study is scarcely comparable to previously published results. The prevalence of RA subjects found in a cohort of 2154 SARS-CoV-2 positive subjects in a large healthcare system in Massachusetts was similar [14], while smaller proportions of autoimmune conditions (with no specification) were found in literature compared to our study [29–32].

### Strengths and limitations

A limitation of the present study is the selection bias due to biased selection of cases and controls, plausible in our situation given that, to date, only symptomatic cases were tested. On the other hand, comparing test-positives with test-negatives we compared subjects with similar characteristics and potentially similar probability of being tested.

One of the strength of this work is the use of the TND, CC-POS and CC-NEG designs, which combined, helped to evaluate potential risk factors for COVID-19 that differ from those for other respiratory infections. Furthermore, the numerousness of the cohorts considered which ensure generalizability of our results.

## Conclusions

Lack of availability of sound scientific knowledge inevitably lead unreliable news to spread over the population, preventing people to disentangle them form reliable information. The rapid circulation of information disseminated in television and social media channels led the World Health Organization to acknowledge that: “We are not just fighting an epidemic, we’re fighting an infodemic” making fake news spread more easily than the virus. Even if additional studies are needed to replicate and strengthen our results, these findings represent initial evidence to derive recommendations based on actual data for subjects with autoimmune diseases.

## Supplementary information

**Additional file 1.** Chronic conditions as classified in the chronic condition administrative database, with source databases and diagnostic codes used, and how a-priori grouped as described in Table 1.

**Additional file 2.** Demographic and clinical characteristics of test-negative matched to test-positive subjects by ASST and date of swab, and population controls for CC-POS and CC-NEG designs.

## Data Availability

Data are not publicly available due to property restrictions.

## Abbreviations

COVID-19: Coronavirus disease 2019
AID: Autoimmune diseases
AHP: Agency for Health Protection
RA: Rheumatoid arthritis
SLE: Systemic lupus erythematosus
GP: General practitioner
RHS: Regional Health System
TND: Test-negative design
CC-POS: Case–control with test-positive as cases
CC-NEG: Case–control with test-negative as cases
OR: Odd ratio
