# Supplementary material for: Association between autoimmune diseases and COVID-19 as assessed in both a test-negative case–control and population case–control design
Source: Auto Immun Highlights. 2020 Oct 6;11(1):15. doi: 10.1186/s13317-020-00141-1 (PMC7537783; doi:10.1186/s13317-020-00141-1)

**Additional File 1**

**Chronic conditions as classified in the chronic condition administrative database, with source databases and diagnostic codes used, and how a-priori grouped as described in Table 1.**

| Comorbidity as in the AHP database | | Comorbidity definition from other AHP databases* | | | | Comorbidity grouping as in Table 1 |
| --- | --- | --- | --- | --- | --- | --- |
| Code | **Description** | **Database** | **Codes (ICD-9-CM/ATC)** | **Yrs** | **Exclusion Criteria** |  |
| 02a | Transplanted-active | Copayment exemption  ODS ‐DRG | 052*  302, 103, 495, 480, 481, 512, 513 | 0‐2  0‐2 |  | Transplanted any time |
| 02b | Transplanted-not active | Copayment exemption  ODS ‐DRG  ODS ‐DiagI and DiagII | 052*  302, 103, 495, 480, 481, 512, 513  V42.0, V42.1, V42.6, V42.7, V42.8*, V42.3, V42.4, V42.5, V42.9 | 2‐10  2‐10  2‐10 | 02a  02a  02a |  |
| 03b | CKD | Copayment exemption  ODS ‐DRG  ODS ‐DiagI and DiagII  Drugs | 023*, 031.403, 031.404  316, 317  V56*, 585*, 586*  V03AE02, V03AE03, V03AE01 | 0‐10  0‐5  0‐5  0‐1 | 03a  03a  03a  03a | CKD |
| 03a | Dialysis dependent CKD | Outpatients | ≥ 70 accesses with code 3995*‐5498* | 0‐1 |  | Dialysis dependent CKD |
| 04 | HIV infection or AIDS | Copayment exemption  Drugs  ODS ‐DRG  ODS ‐DiagI and DiagII | 020*  J05AB14, J05AE*, J05AR*, J05 AG*, J05AX07 (30% DDD)  488, 489, 490  042*, V08 | 0‐10  0‐1  0-5  0-5 |  | HIV infection or AIDS |
| 05a | Tumor in first line treatment | Copayment exemption  Drugs  Drugs  Copayment exemption  ODS ‐DiagI and DiagII  ODS ‐Procedures  Outpatients | 048*  L01* (excluded L01AB01 AND L01AA01)  L01AB01, L01AA01  006.710, 045.696, 028.710, 047.710.1, 046.340  140*‐208*, V580*, V581*  9925*,  9925*, 9224*, 92251*, 92271*, 92273*, 92275*, 92283*, 92284*, 92285*, 92286*, 38991, 3992, 8901M, 897C1, 9229H, 9229J, 9229K, 9229L, 9229M, MAC01, MAC02, MAC03, MAC04 | 0‐1  0‐1  0‐1  0‐1  0‐1  0‐1  0‐1 | Copayment exemptions: 006.710; 045.696; 028.710; 047.710.1; 046.340  Criteria used only if the above criteria are not met | Tumor in first line treatment |
| 05b | Tumor in follow-up, 1-5years | Copayment exemption  ODS ‐DiagI and DiagII  ODS ‐Procedures  Drugs  Drugs  Copayment exemption  Outpatients | 048*  140*‐208*, V580*, V581*  9925*  L02*, L01*(Excluded L01AB01 AND L01AA01)  L01AB01, L01AA01  006.710, 045.696, 028.710, 047.710.1, 046.340  9925*, 9224*, 92251*, 92271*, 92273*, 92275*, 92283*, 92284*, 92285*, 92286*, 38991, 38992, 8901M, 897C1, 9229 H, 9229J, 9229K, 9229L, 9229M, MAC01, MAC02, MAC03, MAC04 | 1‐5  1‐5  1‐5  1‐5  1‐5  1‐5  1‐5 | K05a  K05a  K05a  K05a  K05a; Copayment exemptions:006.710; 045.696; 028.710; 047.710.1; 046.340 between ‐1 and ‐5 yrs;  K05a; Criteria used only if the above criteria are not met | Tumor in follow-up, 1-5years |
| 05b | Tumor in remission after 5 years | Copayment exemption  ODS ‐DiagI and DiagII  ODS ‐Procedures  Drugs  Drugs  Copayment exemption  Outpatients | 048*  140*‐208*, V580*, V581*  9925*  L01*(excluded L01AB0, L01AA01) between ‐5 and -10 yrs  L01AB01, L01AA01  006.710, 045.696, 028.710, 047.710.1, 046.340  9925*, 9224*, 92251*, 92271*, 92273*, 92275*, 92283*, 92284*, 92285*, 92286*, 38991, 38992, 8901M, 897C1, 9229 H, 9229J,9229K,9229L,9229M,MAC01,MAC02, MAC03,MAC04 | 5‐10  5‐10  5‐10  5‐10  5‐10  5‐10  5‐10 | K05b, K05a  K05b, K05a  K05b, K05a  K05b, K05a  K05b, K05a. Copayment exemptions: 006.710;045. 696;028.710; 047.710.1;046.340 Between ‐5 And 10;    K05b, K05a. Criteria used only if the above criteria are not met | Tumor in remission after 5 years† |
| 13b | Tumors | Copayment exemption | Rb* | 0‐10 |  |  |
| 06a | Type 1 diabetes | Copayment exemption  ODS ‐DRG  ODS ‐DiagI and DiagII  Drugs | 013.250 (age at first exemption <35yrs)  295  250.01, 250.03, 250.11, 250.1 3, 250.21, 250.23, 250.31, 250.33  A10A*(Ddd>50%) | 0‐10  0‐5  0‐5  0‐1 |  | Diabetes |
| 06b | Type 2 diabetes | Copayment exemption  ODS ‐DRG  ODS ‐DiagI and DiagII  Drugs | 013.250 (age at first exemption <35yrs)  294  250.00, 250.02, 250.10, 250.1 2, 250.20, 250.22, 250.30, 250.32  A10B* (Ddd>50%) | 0‐10  0‐5  0‐5  0‐1 |  |  |
| 06a | Complicated DM Type 1 | ODS ‐DRG  ODS ‐DiagI and DiagII  Drugs  Outpatients | 285  250.41, 250.43, 250.51, 250.53, 250.61, 250.63, 250.71, 250.73, 250.81, 250.83, 250.91, 2 50.93, 362.01‐363.07, 357.2  N03AX16 OR N03AX12 (Ddd>30%)1433, 1434, 1475, 96591, 96592, 96593, 96594, 96595, 96596 | 0‐5  0‐5  0‐1  0‐1 |  | Complicated DM Type 1 and 2 |
| 06b | Complicated DM Type 2 | ODS ‐DRG  ODS ‐DiagI and DiagII  Drugs  Outpatients | 285  250.42, 250.40, 250.50, 250.52, 250.60, 250.62, 250.70, 20.72, 250.80, 250.82, 250.90, 2 50.92, 362.01‐363.07, 357.2  N03AX16 OR N03AX12 (Ddd>30%)  1433, 1434, 1475, 96591, 96592, 96593, 96594, 96595, 96596 | 0‐5  0‐5  0‐1  0‐1 |  |  |
| 07a | Arterial hypertension | Copayment exemption  Drugs  ODS ‐DRG  ODS ‐DiagI and DiagII | 031.401, 031.405, D31.401, D31.405  C02AC01, C02ca04, C03*, C0 7*, C08c*, C09*(Ddd>50%)  134  401*, 403*, 405* | 0‐10  0‐1  0‐5  0‐5 |  | Arterial hypertension |
| 07b | Ischemic heart disease | Copayment exemption  Drugs  ODS ‐DiagI and DiagII  ODS ‐Procedures | 002.414  C01DA*(Ddd>50%)  410*‐414*  36* | 0‐10  0‐1  0‐5  0‐5 |  | Ischemic heart disease |
| 07b | Valvular heart disease | Copayment exemption  ODS ‐Procedures  ODS ‐DiagI and DiagII | 002.394, 002.395, 002.396, 002.397, 002.424, 002.745, 00 2.746, 002.747, 002.V42.2, 002.V43.335*  394*‐397*, 745*‐747* | 0‐10  0‐5  0‐5 |  | Valvular heart disease |
| 07b | Cardiomyopathy with arrhythmia | Copayment exemption  Drugs  ODS ‐DiagI and DiagII  ODS ‐Procedures  Outpatients | 002.426, 002.427, 002.V45.0  C01b* (Ddd>50%)  426*, 427*, V45.0*  3765, 3766, 3768, 3770, 3787,  3789, 379689481 | 0‐10  0‐1  0‐5  0‐5  0‐1 |  | Cardiomyopathy with arrhythmia |
| 07b | Cardiomyopathy without arrhythmia | Copayment exemption  Drugs  ODS ‐DiagI and DiagII | 031.402, 031.403, 002.416, 002.417, 002.429.4  C02KX01, C02KX02, C02KX03, G04BE03, G04BE08, B01AC09 (Ddd>50%)  402*:404*, 415*, 416*, 417*, 425*, 4294 | 0‐10  0‐1  0‐5 |  | Cardiomyopathy without arrhythmia |
| 07c | Chronic Heart failure | Copayment exemption  ODS ‐DiagI and DiagII  Drugs  Drugs  Drugs | 021.428  428*  C09a*, C09c* (Ddd>50%)  C03ca*, C03cb*, C03eb* (Ddd>50%)  C07ag02, C07ab02, C07ab0 7 (Ddd>50%) | 0‐10  0‐5  0‐1  0‐1  0‐1 | K07c_Atc2, K07_Atc3  K07c_Atc1  K07c_Atc1 | Chronic Hearth failure |
| 13 | Circulatory system disease’ | Copayment exemption | Rg* | 0‐10 |  |  |
| 07b | Peripheral Artery Disease | Copayment exemption  ODS ‐DiagI and DiagII  ODS ‐Procedures | 002.440, 002.441.2, 002.441.4, 002.441.7, 002.441.9, 002.442, 002.444, 002.447.0, 002.447.1, 002.447.6  440*, 441*, 442*, 443.1, 443.2*, 444*, 445*, 447*  3924, 3925, 3926, 3950, 3951, 3952, 3954, 3956, 3957, 3958, 3971, 3972, 3973, 3974, 3979, 3990 | 0‐10  0‐5  0‐5 |  | Peripheral Artery Disease |
| 07b | Venous diseases | Copayment exemption  ODS ‐DiagI and DiagII | 002.452, 002.453, 002.459.1  452*, 453*, 459.1* | 0‐10  0‐5 |  | Venous diseases |
| 07b | Cerebrovascular disease | Copayment exemption  ODS ‐DiagI and DiagII | 002.433, 002.434, 002.437  430*‐438* | 0‐10  0‐5 |  | Cerebrovascular disease |
| 08a | Asthma | Copayment exemption  Drugs  Drugs  ODS ‐DiagI and DiagII | 007.493  R03DC03, R03DC01  R03* (Ddd>30%) Età < 45Aa  493* | 0‐10  0‐1  0‐1  0‐5 |  | Asthma |
| 08b | COPD | Drugs  ODS ‐DiagI and DiagII | R03* (Ddd>30%) Età >=45Aa,  491*, 492*, 494*, 496* | 0‐1  0‐5 |  | COPD |
| 08c | RF or Oxygen therapy | Copayment exemption  ODS ‐DiagI and DiagII  Drugs | 024.518.8, 024.518.81  518.83 518.84  V03AN01 for at least 400 euros | 0‐10  0‐5  0‐1 |  | RF or Oxygen therapy |
| 09a | Chronic hepatitis | Copayment exemption  ODS ‐DiagI and DiagII  Drugs | 016*  070*  L03AB04, L03AB05, L03AB06, L03AB09, L03AB10, L03AB11, L03AB12, L03AB60, L03AB61 (Ddd>50%) | 0‐10  0‐5  0‐1 |  | Chronic hepatitis and cirrhosis |
| 09a | Heatic cirrhosis | Copayment exemption  ODS ‐DiagI and DiagII | 008*  571.2 571.5 571.6571.8, 572.3, 456.0, 456.1, 456.2 | 0‐10  0‐5 |  |  |
| 09a | Chronic Pancreatitis | Copayment exemption  ODS ‐DiagI and DiagII  Drugs | 042*  577.1  A09AA* (Ddd>50%) | 0‐10  0‐5  0‐1 | K05a‐ K05b‐K05c | Digestive system diseases |
| 09a | IBD | Copayment exemption  ODS ‐DiagI and DiagII  Drugs | 009*  555*, 556*  A07EA at least two times | 0‐10  0‐5  0‐1 |  |  |
| 13 | Digestive system diseases, other | Copayment exemption | Ri* | 0‐10 |  |  |
| 10a | Epilepsy | Copayment exemption  Drugs  ODS ‐DiagI and DiagII | 017.345  N03AB02 OR N03AX14 (Ddd>50%)  345* | 0‐10  0‐1  0‐5 |  | Epilepsy |
| 10b | Parkinson and Parkinsonisms | Copayment exemption  Drugs  ODS ‐DiagI and DiagII | 038, 038.332  N04* (Ddd> 30%)  332.0, 332.1 | 0‐10  0‐1  0‐5 |  | Parkinson and Parkinsonisms |
| 10c | Alzheimer | Copayment exemption  Drugs  ODS ‐DiagI and DiagII | 029.331.0  N06D (Ddd >30%)  331.0 | 0‐10  0‐1  0‐5 |  | Alzheimer and Dementias |
| 10f | Dementias | Copayment exemption  ODS ‐DiagI and DiagII | 011.290.0, 011.290.1, 011.290.2, 011.290.4, 011.291.1, 011.290  290*, 294* | 0‐10  0‐5 |  |  |
| 10d | Multiple sclerosis | Copayment exemption  Drugs  ODS ‐DRG  ODS ‐DiagI and DiagII | 046.340  L03AB07, L03AB08, L03AX13, L04AA27, L04AA23  013  340*, 341.9 | 0‐10  0‐10  0‐5  0‐5 |  | Other nervous system diseases |
| 13 | Other diseases of the nervous system | Copayment exemption | Rf* | 0‐10 |  |  |
| 11a | Rheumatoid arthritis | Copayment exemption  ODS ‐DiagI and DiagII | 006*  714.0, 714.1, 714.2, 714.30, 714.32, 714.33 | 0‐10  0‐5 |  | Autoimmune diseases |
| 11b | Systemic lupus erythematosus | Copayment exemption  ODS ‐DiagI and DiagII | 028*  710.0 | 0‐10  0‐5 |  |  |
| 11c | Systemic sclerosis | Copayment exemption  ODS ‐DiagI and DiagII | 047*  710.1 | 0‐10  0‐5 |  |  |
| 11d | Sjogren's disease | Copayment exemption  ODS ‐DiagI and DiagII | 030*  710.2 | 0‐10  0‐5 |  |  |
| 11e | Ankylosing spondylitis | Copayment exemption  ODS ‐DiagI and DiagII | 054*  720.0 | 0‐10  0‐5 |  |  |
| 11f | Myasthenia gravis | Copayment exemption  ODS ‐DiagI and DiagII  Drugs | 034.358.0  358.0  N07AA02 (Ddd>50%) | 0‐10  0‐5  0‐1 |  |  |
| 11h | Autoimmune hemolytic anemias | Copayment exemption  ODS ‐DiagI and DiagII | 003*  283.0 | 0‐10  0‐5 |  |  |
| 11i | Psoriasis and psoriatic arthropathy | Copayment exemption  ODS ‐DiagI and DiagII | 045*  696* | 0‐10  0‐5 |  |  |
| 13m | Connective tissue diseases, others | Copayment exemption | Rm* | 0‐10 |  |  |
| 11g | Hashimoto's thyroiditis | Copayment exemption  ODS ‐DiagI and DiagII | 056*  245.2 | 0‐10  0‐5 |  | Thyroid diseases |
| 12e | Hypothyroidism | Copayment exemption  Drugs  ODS ‐DiagI and DiagII | 027*  H03AA01 (Ddd>30%)  243, 244* | 0‐10  0‐1  0‐5 |  |  |
| 12g | Basedow’s disease | Copayment exemption  ODS ‐DiagI and DiagII | 035*  242.0*, 242.1*, 242.2*, 242.3 | 0‐10  0‐5 |  |  |
| 12a | Acromegaly gigantism | Copayment exemption  Drugs  ODS ‐DiagI and DiagII | 001*  H01CB* (Ddd >50%)  253.0 | 0‐10  0‐1  0‐5 |  | Other endocrine diseases |
| 12b | Diabetes insipidus | Copayment exemption  ODS ‐DiagI and DiagII | 012*  253.5 | 0‐10  0‐5 |  |  |
| 12c | Addison's disease | Copayment exemption  ODS ‐DiagI and DiagII | 022*  255.4 | 0‐10  0‐5 |  |  |
| 12d | Hyper and hypoparathyroidism | Copayment exemption  ODS ‐DiagI and DiagII | 026*  252.0, 252.1 | 0‐10  0‐5 |  |  |
| 12f | Cushing's syndrome | Copayment exemption  ODS ‐DiagI and DiagII | 032*  255.0 | 0‐10  0‐5 |  |  |
| 12h | Pituitary dwarfism | Copayment exemption  ODS ‐DiagI and DiagII | 039*  253.3 | 0‐10  0‐5 |  |  |
| 13c | Other metabolic and endocrine diseases | Copayment exemption | Rc* | 0‐10 |  |  |
| 12i | Familial and sporadic Hypercholesterolemia | Copayment exemption  Drugs  ODS ‐DiagI and DiagII | 025*  C10AA* (Ddd>50%)  272.0, 272.2, 272.4, 272.9 | 0‐10  0‐1  0‐5 |  | Hypercholesterolemia |
| 13d | Blood and Hematopoietic organs | Copayment exemption | Rd* | 0‐10 |  | Blood and Hematopoietic organs |
| 13a | Infectious and parasitic diseases | Copayment exemption | Ra* | 0‐10 |  | Not included |
| 13j | Diseases of the genitourinary system | Copayment exemption | Rj* | 0‐10 |  | Not included |
| 13l | Chronic cutaneous diseases | Copayment exemption | Rl* | 0‐10 |  | Not included |
| 10e | Optic neuromyelitis | Copayment exemption  ODS ‐DiagI and DiagII | 041.341.0  341.0 | 0‐10  0‐5 |  | Not included |
| 13n | Congenital malformation | Copayment exemption | Rn* | 0‐10 |  | Not included |
| 13p | Some perinatal conditions | Copayment exemption | Rp* | 0‐10 |  | Not included |
| 13q | Ill-defined chronic conditions | Copayment exemption | Rq* | 0‐10 |  | Not included |

*for each comorbidity any of the criteria is sufficient

†Moreover, the category 13b called ‘Tumors’ ( (n = 4) was reclassified: 2 cases also had the Tumor in follow-up, 1-5years = 1, the remaining 2 were included in the remission neoplasm = 1

‡ The ‘circulatory system disease’ variable (n = 25) has been merged with the heart failure variable since it is the most frequent outcome of rare circulatory system diseases whose diagnosis is based on the exemption only (not present in the other specific categories)

Abbreviations: CKD=Chronic kidney disease, COPD=Chronic obstructive pulmonary disease, DM=diabetes mellitus, RF=Respiratory Failure

**S2 Pre-specified interactions tested in the model**

| Interaction term | p-value* |
| --- | --- |
| rcs(Age,3)*Gender | 0.0004 |
| CKD*Arterial Hypertension |  |
| CKD* Ischemic heart disease |  |
| CKD*Valvular heart disease |  |
| CKD* Cardiomyopathy with arrhythmia |  |
| CKD* Cardiomyopathy without arrhythmia |  |
| CKD*Chronic heart failure |  |
| CKD* Peripheral Artery Disease |  |
| CKD*Cerebrovascular disease |  |
| Chronic heart failure* Chronic hepatitis and cirrhosis |  |
| COPD*Chronic heart failure |  |
| Gender*Arterial Hypertension |  |
| Gender*Chronic heart failure |  |
| Gender*Complicated Diabetes |  |
| Gender*Diabetes |  |
| Gender* Hypercholesterolemia, familial and non |  |
| rcs(Age,3)*Arterial Hypertension |  |
| rcs(Age,3)*Chronic heart failure | 0.0075 |
| rcs(Age,3)*Complicated DM Type 1 and 2 | <.0001 |
| rcs(Age,3)*COPD |  |
| rcs(Age,3)* Respiratory Failure or Oxygen therapy |  |
| rcs(Age,3)*Diabetes | 0.0021 |
| rcs(Age,3)*Tumor in first line treatment | 0.0019 |

*for significant interactions, included in the final model

CKD=chronic kidney disease, COPD=chronic obstructive pulmonary disease, DM=diabetes mellitus, rcs=restricted cubic spline

Age was introduced in the model as a restricted cubic spline with 3 knots

**S3 Results of the multivariable logistic regression model predicting 30‑days mortality risk from COVID-19 in the development cohort of swab positive cases aged 40 years or older in terms of coefficients, their standard errors (s.e.) and significance (p-values).**

|  | Coefficient | s.e. | p value |
| --- | --- | --- | --- |
| Intercept | -14.26 | 0.82 | <0.0001 |
| rcs(Age, 3)Age | 0.18 | 0.01 | <0.0001 |
| rcs(Age, 3)Age' | -0.11 | 0.01 | <0.0001 |
| Gender | 2.77 | 0.95 | 0.00 |
| Transplanted any time | -0.01 | 0.36 | 0.98 |
| Blood and Hematopoietic organs | -0.91 | 0.78 | 0.25 |
| HIV infection or AIDS | 0.47 | 0.39 | 0.23 |
| Tumor in first line treatment | 5.43 | 1.72 | 0.00 |
| Tumor in follow-up, 1-5years | 0.10 | 0.09 | 0.26 |
| Tumor in remission after 5 years | 0.04 | 0.08 | 0.65 |
| Diabetes (DM) | 4.19 | 1.20 | 0.00 |
| Complicated DM Type 1 and 2 | 10.77 | 2.33 | <0.0001 |
| Familial and sporadic hypercholesterolemia | 0.12 | 0.06 | 0.04 |
| Arterial hypertension | 0.18 | 0.05 | 0.00 |
| Ischemic heart disease | 0.09 | 0.06 | 0.13 |
| Valvular heart disease | 0.02 | 0.11 | 0.83 |
| Cardiomyopathy with arrhythmia | -0.03 | 0.06 | 0.63 |
| Cardiomyopathy without arrhythmia | 0.00 | 0.06 | 0.98 |
| Chronic heart failure | 4.22 | 1.77 | 0.02 |
| Peripheral Artery Disease | 0.03 | 0.10 | 0.75 |
| Venous diseases | 0.26 | 0.17 | 0.13 |
| Cerebrovascular disease | 0.01 | 0.08 | 0.91 |
| Thyroid disease included autoimmune | -0.04 | 0.10 | 0.70 |
| Other endocrine diseases | 0.09 | 0.34 | 0.79 |
| Autoimmune diseases | -0.11 | 0.15 | 0.46 |
| Epilepsy | 0.30 | 0.15 | 0.05 |
| Alzheimer and Dementias | 0.16 | 0.09 | 0.07 |
| Parkinson and Parkinsonisms | 0.06 | 0.13 | 0.67 |
| Nervous system diseases | -0.21 | 0.35 | 0.54 |
| Chronic hepatitis and cirrhosis | 0.12 | 0.20 | 0.54 |
| Digestive system, other | -0.20 | 0.20 | 0.32 |
| Chronic obstructive pulmonary disease | 0.00 | 0.08 | 1.00 |
| Respiratory Failure or Oxygen therapy | 0.21 | 0.23 | 0.37 |
| Asthma | 0.04 | 0.16 | 0.79 |
| Chronic kidney disease (CKD) | 0.19 | 0.10 | 0.05 |
| Dialysis dependent CKD | 0.42 | 0.19 | 0.03 |
| rcs(Age, 3)Age:Gender | -0.03 | 0.01 | 0.02 |
| rcs(Age, 3)Age':Gender | 0.04 | 0.01 | 0.00 |
| rcs(Age, 3)Age:Chronic heart failure | -0.06 | 0.03 | 0.04 |
| rcs(Age, 3)Age':Chronic heart failure | 0.03 | 0.02 | 0.16 |
| rcs(Age, 3)Age:Complicated DM Type 1 and 2 | -0.16 | 0.04 | <0.0001 |
| rcs(Age, 3)Age':Complicated DM Type 1 and 2 | 0.14 | 0.03 | <0.0001 |
| rcs(Age, 3)Age:Diabetes | -0.06 | 0.02 | 0.00 |
| rcs(Age, 3)Age':Diabetes | 0.04 | 0.02 | 0.01 |
| rcs(Age, 3)Age:Tumor in first line treatment | -0.08 | 0.03 | 0.00 |
| rcs(Age, 3)Age':Tumor in first line treatment | 0.05 | 0.02 | 0.04 |

**S 4 Distribution of predictors of 30-days mortality from COVID-19 in the population 40 year-old or older residing in the Agency for Health Protection of Milan territory**

| Predictor | Overall |
| --- | --- |
|  | n=2,233,453 |
| Gender = Male (%) | 1048573 (46.9) |
| Age class (years) (%) |  |
| 40-59 | 1150795 (51.5) |
| 60-79 | 780764 (35.0) |
| 80+ | 301894 (13.5) |
| Trasplanted any time = Yes (%) | 5121 ( 0.2) |
| Blood and Hematopoietic organs = Yes (%) | 3122 ( 0.1) |
| HIV infection or AIDS = Yes (%) | 10345 ( 0.5) |
| Tumor in first line treatment = Yes (%) | 92316 ( 4.1) |
| Tumor in follow-up, 1-5years = Yes (%) | 68792 ( 3.1) |
| Tumor in remission after 5 years = Yes (%) | 93295 ( 4.2) |
| Type 1 Diabetes = Yes (%) | 2200 ( 0.1) |
| Type 2 Diabetes = Yes (%) | 169277 ( 7.6) |
| Complicated DM Type 1 and 2 = Yes (%) | 15119 ( 0.7) |
| Familial and non hypercholesterolaemia = Yes (%) | 206436 ( 9.2) |
| Arterial hypertension = Yes (%) | 674788 (30.2) |
| Ischemic heart disease = Yes (%) | 132805 ( 5.9) |
| valvular heart disease = Yes (%) | 28082 (1.3) |
| Cardiomyopathy with arrhythmia = Yes (%) | 114494 ( 5.1) |
| Cardiomyopathy without arrhythmia = Yes (%) | 96648 ( 4.3) |
| Chronic hearth failure = Yes (%) | 56986 ( 2.6) |
| Peripheral Artery Disease = Yes (%) | 26873 ( 1.2) |
| Venous diseases = Yes (%) | 12403 ( 0.6) |
| Cerebrovascular disease = Yes (%) | 28413 ( 1.3) |
| Tyroid diseases = Yes (%) | 123570 ( 5.5) |
| Other endocrin diseases = Yes (%) | 6948 ( 0.3) |
| Other autoimmune diseases= Yes (%) | 32627 ( 1.5) |
| Epilepsy = Yes (%) | 14542 ( 0.7) |
| Alzheimer and Dementias = Yes (%) | 14607 ( 0.7) |
| Parkinson and Parkinsonisms = Yes (%) | 11406 ( 0.5) |
| Nervous system diseases, others = Yes (%) | 9414 ( 0.4) |
| Chronic hepatitis and cirrhosis = Yes (%) | 38537 ( 1.7) |
| Digestive system diseases, others = Yes (%) | 22288 ( 1.0) |
| Chronic obstructive pulmonary disease = Yes (%) | 46643 ( 2.1) |
| Respiratory Failure or Oxygen therapy = Yes (%) | 3680 ( 0.2) |
| Asthma = Yes (%) | 40450 ( 1.8) |
| CKD = Yes (%) | 26495 ( 1.2) |
| Dialysis dependent CKD = Yes (%) | 2687 ( 0.1) |

**S5 Results of the multivariable logistic regression model predicting 30‑days mortality risk from COVID-19 in the swab positive cases of all ages in terms of coefficients, their standard errors (s.e.) and significance (p-values).**

|  | Coefficient | s.e. | p value |
| --- | --- | --- | --- |
| Intercept | -15.44 | 0.96 | <0.0001 |
| rcs(Age, 3)Age | 0.21 | 0.02 | <0.0001 |
| rcs(Age, 3)Age' | -0.11 | 0.01 | <0.0001 |
| Gender | 4.04 | 1.11 | 0.00 |
| Trasplanted any time | 0.09 | 0.35 | 0.80 |
| Blood and Hematopoietic organs | -0.87 | 0.78 | 0.26 |
| HIV infection or AIDS | 0.44 | 0.38 | 0.25 |
| Tumor in first line treatment | 6.11 | 1.74 | 0.00 |
| Tumor in follow-up, 1-5years | 0.11 | 0.09 | 0.23 |
| Tumor in remission after 5 years | 0.04 | 0.08 | 0.62 |
| Diabetes (DM) | 4.31 | 1.36 | 0.00 |
| Complicated DM Type 1 and 2 | 12.63 | 2.78 | <0.0001 |
| Familial and non hypercholesterolaemia | 0.13 | 0.06 | 0.03 |
| Arterial hypertension | 0.18 | 0.05 | 0.00 |
| Ischemic heart disease | 0.09 | 0.06 | 0.13 |
| valvular heart disease | 0.03 | 0.11 | 0.82 |
| Cardiomyopathy with arrhythmia | -0.03 | 0.06 | 0.63 |
| Cardiomyopathy without arrhythmia | 0.00 | 0.06 | 0.96 |
| Chronic hearth failure | 4.15 | 2.14 | 0.05 |
| Peripheral Artery Disease | 0.04 | 0.10 | 0.71 |
| Venous diseases | 0.26 | 0.17 | 0.13 |
| Cerebrovascular disease | 0.02 | 0.08 | 0.86 |
| Tyroid disease included autoimmune | -0.04 | 0.10 | 0.67 |
| Other endocrin diseases | 0.11 | 0.34 | 0.75 |
| Autoimmune diseases | -0.11 | 0.15 | 0.47 |
| Epilepsy | 0.31 | 0.15 | 0.04 |
| Alzheimer and Dementias | 0.17 | 0.09 | 0.06 |
| Parkinson and Parkinsonisms | 0.07 | 0.13 | 0.59 |
| Nervous system diseases | -0.21 | 0.35 | 0.55 |
| Chronic hepatitis and cirrhosis | -0.08 | 0.13 | 0.55 |
| Digestive system, other | -0.20 | 0.20 | 0.32 |
| Chronic obstructive pulmonary disease | 0.01 | 0.08 | 0.93 |
| Respiratory Failure or Oxygen therapy | 0.20 | 0.23 | 0.38 |
| Asthma | 0.04 | 0.16 | 0.78 |
| Chronic kidney disease (CKD) | 0.19 | 0.10 | 0.05 |
| Dialysis dependent CKD | 0.42 | 0.19 | 0.03 |
| rcs(Age, 3)Age:Gender | -0.06 | 0.02 | 0.00 |
| rcs(Age, 3)Age':Gender | 0.05 | 0.01 | <0.0001 |
| rcs(Age, 3)Age:Chronic hearth failure | -0.06 | 0.03 | 0.10 |
| rcs(Age, 3)Age':Chronic hearth failure | 0.03 | 0.02 | 0.28 |
| rcs(Age, 3)Age:Complicated DM Type 1 and 2 | -0.20 | 0.05 | <0.0001 |
| rcs(Age, 3)Age':Complicated DM Type 1 and 2 | 0.14 | 0.04 | <0.0001 |
| rcs(Age, 3)Age:Diabetes | -0.06 | 0.02 | 0.00 |
| rcs(Age, 3)Age':Diabetes | 0.04 | 0.02 | 0.02 |
| rcs(Age, 3)Age:Tumor in first line treatment | -0.09 | 0.03 | 0.00 |
| rcs(Age, 3)Age':Tumor in first line treatment | 0.05 | 0.02 | 0.02 |

**Supplementary figures**

**Figure S1 Development and external validation cohorts**

**
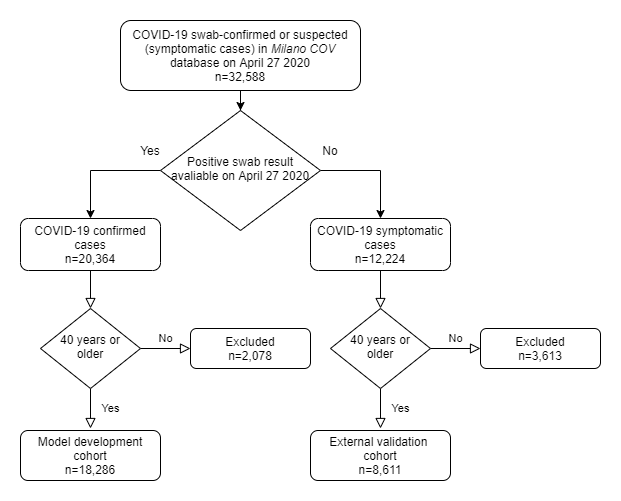
**

**Figure S2 Calibration plot of the multivariable logistic regression model predicting 30‑days mortality risk from COVID-19 in the development cohort of swab positive cases aged 40 years or older**

**
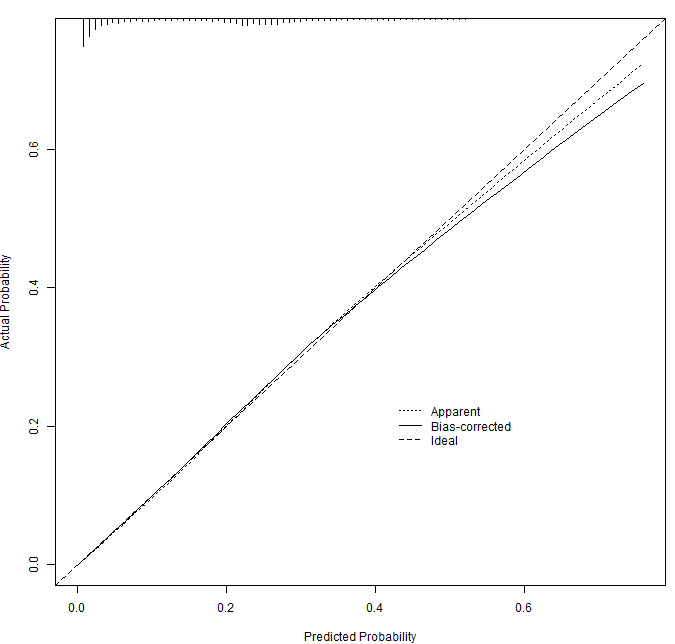
**

**Figure S3 Calibration plot of the multivariable logistic regression model predicting 30‑days mortality risk from COVID-19 in the external validation cohort of symptomatic cases aged 40 years or older**


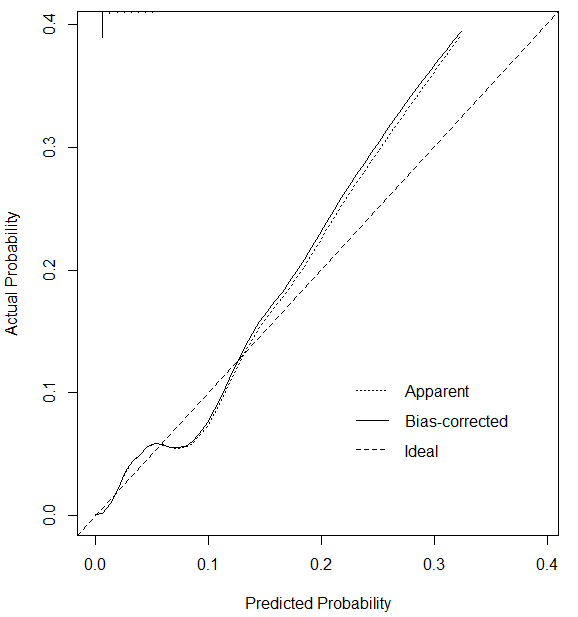

Supplement: Supplementary file 1 — Additional file 1. Chronic conditions as classified in the chronic condition administrative database, with source databases and diagnostic codes used, and how a-priori grouped as described in Table 1. [file 13317_2020_141_MOESM1_ESM.docx]
