# Supplementary material for: Association between autoimmune diseases and COVID-19 as assessed in both a test-negative case–control and population case–control design
Source: Auto Immun Highlights. 2020 Oct 6;11(1):15. doi: 10.1186/s13317-020-00141-1 (PMC7537783; doi:10.1186/s13317-020-00141-1)
Supplement: Supplementary file 2 — Additional file 2. Demographic and clinical characteristics of test-negative matched to test-positive subjects by ASST and date of swab, and population controls for CC-POS and CC-NEG designs. [file 13317_2020_141_MOESM2_ESM.docx]

Additional file 2. Demographic and clinical characteristics of test-negative matched to test-positive subjects by ASST and date of swab, and population controls for CC-POS and CC-NEG designs.

|  | Test-negative subjects*  (for TND and CC-NEG designs) | | | Controls  (for CC-POS and CC-NEG designs) | | |
| --- | --- | --- | --- | --- | --- | --- |
|  |  | Autoimmune  status | |  | Autoimmune  status | |
|  | **Overall** | **No** | **Overall** | **Overall** | **No** | **Yes** |
|  | 19251 | n= 18522; 96.2% | n= 729; 3.8% | n=154008 | n=150626; 97.8% | n=3382; 2.2% |
| Gender = Male (%) | 7650 (39.7) | 7538 (40.7) | 112 (15.4) | 74542 (48.4) | 73904 (49.1) | 638 (18.9) |
| Age mean (sd) | 54.8 (20.6) | 54.7 (20.7) | 56.6 (16) | 45.4 (23.7) | 45.2 (23.7) | 58.4 (16.2) |
| Age class (years) (%) |  |  |  |  |  |  |
| <17 | 504 (2.6) | 503 (2.7) | 1 (0.1) | 24652 (16) | 24630 (16.4) | 22 (0.7) |
| [18-40) | 4081 (21.2) | 3987 (21.5) | 94 (12.9) | 35439 (23) | 35022 (23.3) | 417 (12.3) |
| [40-70) | 9645 (50.1) | 9180 (49.6) | 465 (63.8) | 66030 (42.9) | 64049 (42.5) | 1981 (58.6) |
| ≥70 | 5021 (26.1) | 4852 (26.2) | 169 (23.2) | 27887 (18.1) | 26925 (17.9) | 962 (28.4) |
| Geographic location (%) |  |  |  |  |  |  |
| Lodi Province (start of the outburst) | 2537 (13.2) | 2452 (13.2) | 85 (11.7) | 10050 (6.5) | 9800 (6.5) | 250 (7.4) |
| City of Milan | 7057 (36.7) | 6825 (36.8) | 232 (31.8) | 60725 (39.4) | 59484 (39.5) | 1241 (36.7) |
| Milan Province | 9657 (50.2) | 9245 (49.9) | 412 (56.5) | 83233 (54) | 81342 (54) | 1891 (55.9) |
| Number of Comorbidities  (Excluded autoimmune) |  |  |  |  |  |  |
| None |  |  |  | 10536 (68.4) | 10444 (69.3) | 914 (27) |
| 1-3 | 10330 (53.7) | 10100 (54.5) | 230 (31.6) | 41971 (27.3) | 39881 (26.5) | 2090 (61.8) |
| ≥4 | 6740 (35) | 6352 (34.3) | 388 (53.2) | 6674 (4.3) | 6296 (4.2) | 378 (11.2) |
| Comorbidities (%) | 2181 (11.3) | 2070 (11.2) | 111 (15.2) |  |  |  |
| Transplanted any time = Yes (%) |  |  |  | 245 (0.2) | 238 (0.2) | 7 (0.2) |
| Blood and Hematopoietic organs = Yes (%) | 108 (0.6) | 106 (0.6) | 2 (0.3) | 180 (0.1) | 169 (0.1) | 11 (0.3) |
| HIV infection or AIDS = Yes (%) | 36 (0.2) | 31 (0.2) | 5 (0.7) | 510 (0.3) | 502 (0.3) | 8 (0.2) |
| Tumor in first line treatment = Yes (%) | 125 (0.6) | 122 (0.7) | 3 (0.4) | 4153 (2.7) | 3468 (2.3) | 685 (20.3) |
| Tumor in follow-up, 1-5years = Yes (%) | 1204 (6.3) | 1108 (6) | 96 (13.2) | 3182 (2.1) | 3077 (2) | 105 (3.1) |
| Tumor in remission after 5 years = Yes (%) | 622 (3.2) | 593 (3.2) | 29 (4) | 4141 (2.7) | 3951 (2.6) | 190 (5.6) |
| Type 1 Diabetes = Yes (%) | 744 (3.9) | 711 (3.8) | 33 (4.5) | 185 (0.1) | 173 (0.1) | 12 (0.4) |
| Type 2 Diabetes = Yes (%) | 45 (0.2) | 43 (0.2) | 2 (0.3) | 7527 (4.9) | 7269 (4.8) | 258 (7.6) |
| Complicated DM Type 1 and 2 = Yes (%) | 1400 (7.3) | 1341 (7.2) | 59 (8.1) | 637 (0.4) | 616 (0.4) | 21 (0.6) |
| Hypercholesterolaemia = Yes (%) | 313 (1.6) | 296 (1.6) | 17 (2.3) | 8952 (5.8) | 8590 (5.7) | 362 (10.7) |
| Arterial hypertension = Yes (%) | 1509 (7.8) | 1450 (7.8) | 59 (8.1) | 28928 (18.8) | 27761 (18.4) | 1167 (34.5) |
| Ischemic heart disease = Yes (%) | 5336 (27.7) | 5098 (27.5) | 238 (32.6) | 5654 (3.7) | 5468 (3.6) | 186 (5.5) |
| Valvular heart disease = Yes (%) | 1515 (7.9) | 1451 (7.8) | 64 (8.8) | 1394 (0.9) | 1347 (0.9) | 47 (1.4) |
| Cardiomyopathy with arrhythmia = Yes (%) | 384 (2) | 365 (2) | 19 (2.6) | 4940 (3.2) | 4754 (3.2) | 186 (5.5) |
| Cardiomyopathy without arrhythmia = Yes (%) | 1442 (7.5) | 1384 (7.5) | 58 (8) | 4072 (2.6) | 3932 (2.6) | 140 (4.1) |
| Chronic Hearth failure = Yes (%) | 1137 (5.9) | 1089 (5.9) | 48 (6.6) | 2375 (1.5) | 2289 (1.5) | 86 (2.5) |
| Peripheral Artery Disease = Yes (%) | 1001 (5.2) | 957 (5.2) | 44 (6) | 1158 (0.8) | 1108 (0.7) | 50 (1.5) |
| Venous diseases = Yes (%) | 463 (2.4) | 444 (2.4) | 19 (2.6) | 551 (0.4) | 527 (0.3) | 24 (0.7) |
| Cerebrovascular disease = Yes (%) | 170 (0.9) | 159 (0.9) | 11 (1.5) | 1168 (0.8) | 1100 (0.7) | 68 (2) |
| Thyroid diseases = Yes (%) | 493 (2.6) | 471 (2.5) | 22 (3) | 5890 (3.8) | 3767 (2.5) | 2123 (62.8) |
| Other endocrine diseases = Yes (%) | 1225 (6.4) | 789 (4.3) | 436 (59.8) | 495 (0.3) | 458 (0.3) | 37 (1.1) |
| Other autoimmune diseases= Yes (%) | 98 (0.5) | 83 (0.4) | 15 (2.1) | 1540 (1) | 107 (0.1) | 1433 (42.4) |
| Epilepsy = Yes (%) | 350 (1.8) | 28 (0.2) | 322 (44.2) | 847 (0.5) | 815 (0.5) | 32 (0.9) |
| Alzheimer and Dementias = Yes (%) | 220 (1.1) | 214 (1.2) | 6 (0.8) | 657 (0.4) | 640 (0.4) | 17 (0.5) |
| Parkinson and Parkinsonisms = Yes (%) | 311 (1.6) | 302 (1.6) | 9 (1.2) | 486 (0.3) | 467 (0.3) | 19 (0.6) |
| Nervous system diseases, others = Yes (%) | 163 (0.8) | 160 (0.9) | 3 (0.4) | 519 (0.3) | 495 (0.3) | 24 (0.7) |
| Chronic hepatitis and cirrhosis = Yes (%) | 124 (0.6) | 116 (0.6) | 8 (1.1) | 1764 (1.1) | 1687 (1.1) | 77 (2.3) |
| Digestive system diseases, others = Yes (%) | 407 (2.1) | 381 (2.1) | 26 (3.6) | 1350 (0.9) | 1272 (0.8) | 78 (2.3) |
| COPD = Yes (%) | 259 (1.3) | 244 (1.3) | 15 (2.1) | 1987 (1.3) | 1901 (1.3) | 86 (2.5) |
| RF or Oxygen therapy = Yes (%) | 631 (3.3) | 598 (3.2) | 33 (4.5) | 160 (0.1) | 153 (0.1) | 7 (0.2) |
| Asthma = Yes (%) | 89 (0.5) | 84 (0.5) | 5 (0.7) | 2608 (1.7) | 2505 (1.7) | 103 (3) |
| CKD = Yes (%) | 476 (2.5) | 443 (2.4) | 33 (4.5) | 1209 (0.8) | 1166 (0.8) | 43 (1.3) |
| Dialysis dependent CKD = Yes (%) | 423 (2.2) | 404 (2.2) | 19 (2.6) | 104 (0.1) | 103 (0.1) | 1 (0) |
| Autoimmune disease | 254 (1.3) | 246 (1.3) | 8 (1.1) |  |  |  |
| Acquired Autoimmune Hemolytic Anemia |  |  |  |  |  | 17 (0.5) |
| Systemic Sclerosis |  |  | 7 (1) |  |  | 77 (2.3) |
| Ankylosing Spondylitis |  |  | 25 (3.4) |  |  | 51 (1.5) |
| Myasthenia Gravis |  |  | 11 (1.5) |  |  | 46 (1.4) |
| Rheumatoid Arthritis |  |  | 17 (2.3) |  |  | 578 (17.1) |
| Psoriatic Arthritis |  |  | 141 (19.3) |  |  | 392 (11.6) |
| Systemic Lupus Erythematosus |  |  | 73 (10) |  |  | 109 (3.2) |
| Sjogren Disease |  |  | 22 (3) |  |  | 100 (3) |
| Hashimoto's Disease |  |  | 29 (4) |  |  | 2012 (59.5) |
| *Test-negative matched to test-positive subjects by ASST and date of swab. | | | | | | |
